# Urban land cover type determines the sensitivity of carbon dioxide fluxes to precipitation in Phoenix, Arizona

**DOI:** 10.1371/journal.pone.0228537

**Published:** 2020-02-12

**Authors:** Elí R. Pérez-Ruiz, Enrique R. Vivoni, Nicole P. Templeton

**Affiliations:** 1 School of Earth and Space Exploration, Arizona State University, Tempe, AZ, United States of America; 2 Departamento de Ingeniería Civil y Ambiental, Instituto de Ingeniería y Tecnología, Universidad Autónoma de Ciudad Juárez, Ciudad Juárez, México; 3 School of Sustainable Engineering and the Built Environment, Arizona State University, Tempe, AZ, United States of America; 4 Holistic Engineering and Land Management, Phoenix, AZ, United States of America; University of California Santa Barbara, UNITED STATES

## Abstract

Urbanization modifies land surface characteristics with consequent impacts on local energy, water, and carbon dioxide (CO_2_) fluxes. Despite the disproportionate impact of cities on CO_2_ emissions, few studies have directly quantified CO_2_ conditions for different urban land cover patches, in particular for arid and semiarid regions. Here, we present a comparison of eddy covariance measurements of CO_2_ fluxes (*FC*) and CO_2_ concentrations ([CO_2_]) in four distinct urban patches in Phoenix, Arizona: a xeric landscaping, a parking lot, a mesic landscaping, and a suburban neighborhood. Analyses of diurnal, daily, and seasonal variations of *FC* and [CO_2_] were related to vegetation activity, vehicular traffic counts, and precipitation events to quantify differences among sites in relation to their urban land cover characteristics. We found that the mesic landscaping with irrigated turf grass was primarily controlled by plant photosynthetic activity, while the parking lot in close proximity to roads mainly exhibited the signature of vehicular emissions. The other two sites that had mixtures of irrigated vegetation and urban surfaces displayed an intermediate behavior in terms of CO_2_ fluxes. Precipitation events only impacted *FC* in urban patches without outdoor water use, indicating that urban irrigation decouples CO_2_ fluxes from the effects of infrequent storms in an arid climate. These findings suggest that the proportion of irrigated vegetation and urban surfaces fractions within urban patches could be used to scale up CO_2_ fluxes to a broader city footprint.

## Introduction

Urbanization modifies land surface characteristics and impacts local energy, water, and carbon dioxide (CO_2_) fluxes, particularly when large changes are made as compared to pre-existing conditions [1–9]. Cities are the most visible sign of global change and, despite their relatively small global areal fraction (2 to 5%), urban areas are responsible for >70% of the total CO_2_ emissions from anthropogenic sources [6, 10–16]. In arid and semiarid regions, the trend in urbanization is even more pronounced than in other climate settings, which is crucial since about 30% of the global population is currently residing in cities in arid and semiarid climates [17–19]. Furthermore, prior efforts have shown that urbanization in these regions significantly impacts CO_2_ concentrations and emissions estimated for different land cover types [3, 16, 20, 21].

CO_2_ fluxes in urban areas are influenced by anthropogenic emissions, vegetation, and hydrometeorological factors such as precipitation and temperature. Most CO_2_ fluxes in cities are controlled by fuel combustion from vehicles, industries and buildings, rather than by biological processes [6, 10, 22–27]. As a result, urban areas are net sources of CO_2_ to the atmosphere [6, 28–30], though a high degree of spatiotemporal heterogeneity is present [6, 15, 31–33]. Furthermore, the influence of point sources of CO_2_ can play a disproportionate role as compared to natural ecosystems [6, 34]. Nevertheless, it is possible that urban vegetation can potentially have an important role in modulating CO_2_ exchanges in cities by counteracting to some extent those positive fluxes through the photosynthetic activity of plants. This urban vegetation effect is modulated by the amount of available water, provided in arid and semiarid cities through irrigation [7, 35, 36], and the availability of incoming solar radiation affected by cloud cover. The competing effects of anthropogenic emissions (sources) and plant-mediated CO_2_ fixation (sinks) in arid cities are not well understood at present [9, 16, 25, 30, 37–39].

A number of approaches exist to estimate CO_2_ exchanges in cities, including bottom-up methods using emission factors, indirect calculations through CO_2_ concentrations [38, 40–42], and CO_2_ inverse modeling [43, 44]. Indirect methods such as these are often associated with large uncertainties and a lack of spatial and temporal detail [6]. As an alternative applied in this study, the eddy covariance (EC) method [45] can be used to measure CO_2_ fluxes (*FC*) in urban areas [6, 11, 12, 15, 16, 30, 39, 46]. However, urban EC observations have been generally limited, as compared to those efforts in natural ecosystems, with most studies undertaken in very dense urban settings or in open low-density areas of northern latitudes [6, 47–53]. Furthermore, arid cities have been generally underrepresented in the use of the EC method [16, 25]. This paucity of studies is related to the challenging nature of urban *FC* observations due to deployment logistics, security concerns, and the potential disruption of activities [49, 54]. As the number of EC studies in urban areas grows, however, it will be possible to assemble inventories of CO_2_ flux measurements that can be compared to bottom up approaches.

Limitations in urban EC studies also imply that few efforts have been carried out to quantify the role of land cover type on *FC* measurements, for instance between urban parks and the high-density urban core. Relevant measurements represent a challenge due the spatial variability of urban land covers and the complex morphology of urban environments [12, 16, 44]. Several studies have measured CO_2_ exchanges in urban areas relative their surrounding environments. For example, *Bergeron and Strachan* [30] compared agricultural, suburban, and urban sites near Montreal, Canada. *Ward et al*. [55] similarly studied three areas (woodland, suburban, and urban sites) in England, while *Buckley et al*. [13] compared *FC* measurements in suburban and urban sites in Syracuse, USA. *Ueyama and Ando* [15] is one of the few studies to perform a direct comparison of multiple urban patches in Japan. In Indianapolis, as part of the INFLUX experiment, an important effort to measure *FC* over several urban landscapes was also carried out [56, 57]. However, arid cities are under-represented in terms of *FC* measurements with the EC method, though *Song et al*. [16] analyzed conditions in Phoenix, USA.

In this study, we use a mobile EC tower to measure *FC* and meteorological conditions in three urban settings at Arizona State University (ASU) as described by *Templeton et al*. [58] and similar to *Soegaard and Møller-Jensen* [59]. These short-term deployments are compared to a stationary (reference) EC tower in a suburban neighborhood and spanning the entire period (1 January to 30 September 2015). The three mobile sites represent different land cover types: a xeric landscaping, a parking lot, and a mesic landscaping. These sites are expected to vary in terms of their CO_2_ exchanges due to variations in the amount of vegetation and anthropogenic emissions. Thus, the objectives of this study are to: (1) quantify and compare *FC* over different urban land cover types in relation to a location that provided reference meteorological conditions during the study period, (2) relate the observed differences to measures of anthropogenic emissions, plant photosynthetic activity, and meteorological forcing, and (3) determine the role of precipitation events and outdoor water use on modifying CO_2_ exchanges across the sites.

## Materials and methods

### Site descriptions

The study was carried out in four locations in the Phoenix Metropolitan Area (PMA) as described in Table 1 that were non overlapping and at most 42.8 km apart. The PMA has a population of 4.1 million [60] and is located in a hot, arid climate (Köppen classification BWh), with seasonal average temperatures of 14.1°C, 22.9°C, 33.9°C, and 24.8°C, in the winter, spring, summer, and fall. A bimodal precipitation regime is present with winter frontal storms and summer thunderstorms during the North American monsoon [61, 62]. Mean annual precipitation is 204 mm yr^-1^ based on 1981 to 2010 data, with winter (December to January) and summer (July to September) amounts of 68.3 mm and 67.8 mm, respectively. Spring and early summer (March through June) are typically dry, accounting for only 17% of the mean annual precipitation [7, 58]. The low annual precipitation leads to water limited conditions in natural ecosystems [62], requiring outdoor water use to support vegetation in urban areas [7, 35, 36].

**Table 1:** General characteristics of the four study sites and sampling periods.

Site	Land Cover	Latitude	Longitude	Elevation (m)	Start Day and Time	End Day and Time	Total Days
**XL**	Xeric Landscaping	33.4198°	-111.9272°	354	1/20/2015 12:00	3/13/2015 8:30	53
**PL**	Pavement	33.4212°	-111.9387°	356	5/19/2015 15:00	6/30/2015 6:00	43
**ML**	Mesic Landscaping	33.3116°	-111.6806°	411	7/9/2015 13:00	9/18/2015 8:30	72
**REF**	Residential	33.4838°	-112.1426°	337	1/1/2015 0:00	10/13/2015 23:30	286

**Fig 1:**
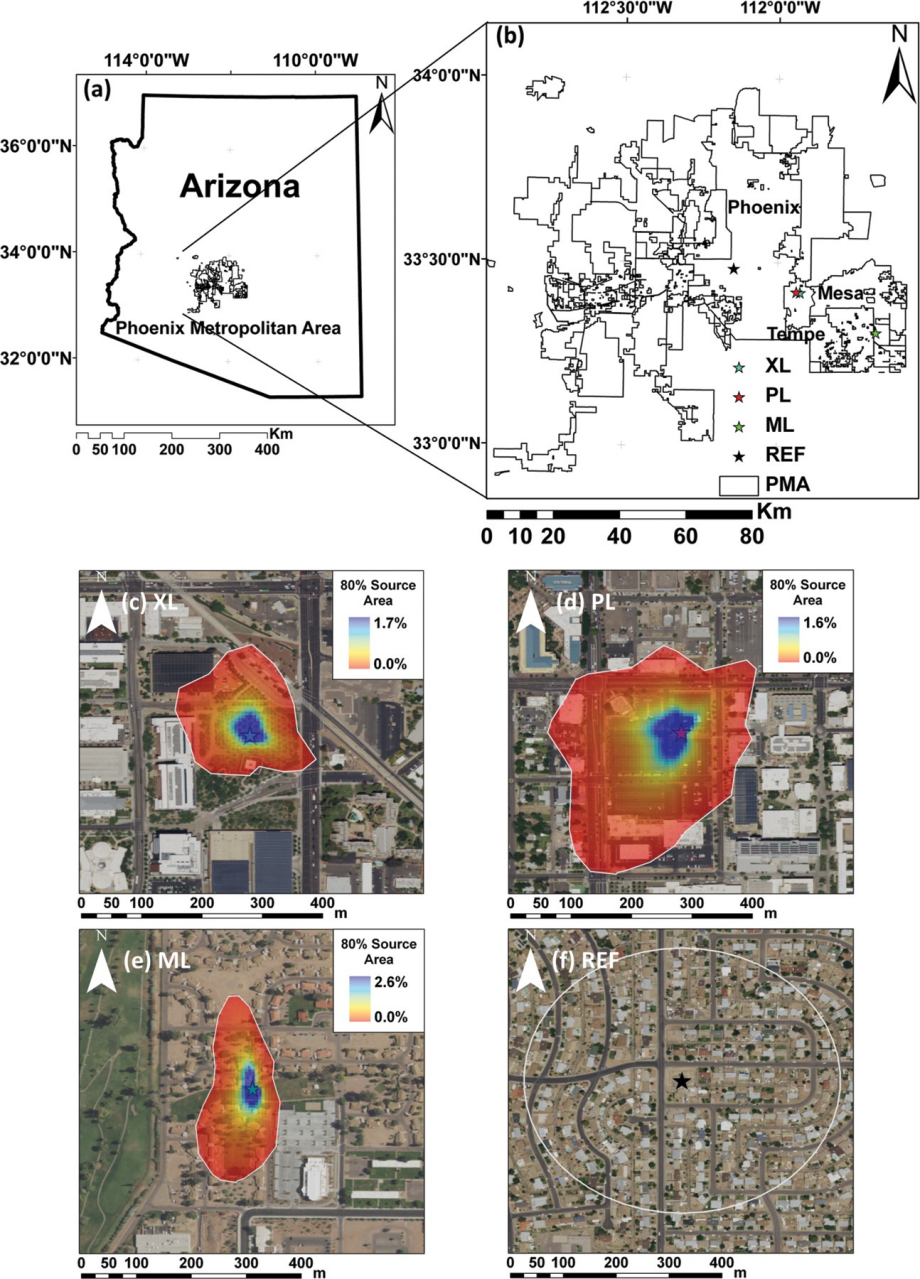
Location of the study sites. (a) Location of the Phoenix Metropolitan Area, and (b) the four study sites: (c) xeric landscaping (XL) site at ASU Tempe campus; (d) parking lot (PL) site near a high traffic intersection at ASU Tempe campus; (e) mesic landscaping (ML) site near residential housing at ASU Polytechnic campus; and (f) suburban (REF) site in Phoenix. Contour polygon and color gradient represent the 80% cumulative source area for each site. Aerial images of the sites correspond to National Agricultural Imagery Program (NAIP) from the U.S. Geological Survey (https://doi.org/10.5066/F7QN651G).

The three mobile deployments and the reference site represent different urban land covers in the PMA. Fig 1 presents an aerial image of each sampling location that depicts differences in urban characteristics. These urban land covers correspond to: (a) xeric landscaping (XL) site, classified as a Local Climate Zone (LCZ) 5 [63] composed of drip-irrigated trees (palo verde, *Parkinsonia florida*) of 3–4 m of height, with gravel and bare soil cover, located within a setting that included a midrise (three-story) building used for office space and a paved road; (b) a parking lot (PL) site, classified as a LCZ 8 [63], characterized by pavement (asphalt) with minimal vegetation, near an intersection with high traffic and frequently contained vehicles, with a low number of 6 m palm trees and large low-rise (one- to three-story) buildings used for office space surrounded by impervious cover nearby; (c) a mesic landscaping (ML), classified as LCZ 9, consisting of a sprinkler-irrigated turf grass (approximately 2–3 days per week, 3 times per day, for 20 to 30 min each time) among sparsely built single-family homes (low-rise, one story) with sparse, undeveloped land cover nearby including sparse 6 m trees; and (d) a suburban residential area, classified as LCZ 6, consisting of medium-density single-family homes, streets, and open spaces, used as a reference site (REF). As compared to ML, the REF site has lower irrigation due to larger variations in landscaping with some yards having trees and grasses, but most containing gravel and bare soil. One of the sites (PL) is nearly devoid of vegetation, while one site (ML) has light traffic. The REF site is a stationary EC system in operation during the entire sampling period and spanning the seasonal changes in meteorological conditions to allow quantitative comparisons with the short-term deployments. All mobile deployments were within ASU (Tempe campus for XL and PL and Polytechnic campus for ML) and authorized through the ASU Facilities Department.

A land cover classification was performed for the three mobile deployments using color (0.30 m) orthoimagery from the U.S. Geological Survey (http://lta.cr.usgs.gov/high_res_ortho). A supervised classification based on RGB signatures was done using a maximum likelihood method and classifying the urban land cover as (1) trees, (2) grass, (3) undeveloped (gravel or bare soil), (4) pavement (asphalt), and (5) buildings or concrete. Percentages of each land cover type within a unique EC footprint were derived from aggregations of 30-min interval daytime footprint estimates. The EC footprint was obtained using the analytical model of *Kormann and Meixner* [64] for an area of 500 m by 500 m centered at each site and a horizontal pixel resolution of 5 m selected to be less than the measurement height [65]. Following *Anderson and Vivoni* [66], the EC footprint [67] was calculated for each 30 min interval of turbulent daytime conditions, averaged over each daytime period and aggregated to derive a unique footprint for each deployment. The proportion of land cover in the 80% cumulative source area around each deployment can be seen in Table 2. *Chow et al*. [7] determined the land cover at the REF site based on a 2.4 m resolution Quickbird image for a circular region of 1 km^2^ around the location.

**Table 2:** Urban land cover percentages for each site, with REF reported by *Chow et al*. [7].

Urban Land Cover	80% Cumulative Source Area			
	XL	PL	ML	REF
Trees	38.2%	5.9%	16.2%	4.6%
Grass	0.4%	0.7%	28.1%	10.0%
Undeveloped	29.7%	13.9%	34.6%	36.8%
Pavement	8.3%	57.4%	12.8%	22.0%
Buildings or concrete	23.4%	22.1%	8.3%	26.4%

At the XL site, the 80% cumulative footprint is influenced mainly (Fig 1C) by the 3–4 m trees around the tower, with some contributions from a street to the east, a public transportation center to the north, and a minimum impact of a three-story building to the west. The PL footprint is influenced primarily by the parking surface and two nearby streets to the north and west, with limited influence from surrounding buildings. At the ML site, the irrigated turf grass around the tower is the main contributor and to a lesser extent there is an influence of sparse trees and one-story houses. The highest vegetation cover is present at ML (44.3%), while the lowest occurs at PL (6.6%). A large contrast is also present in the areal coverage of urban surfaces (buildings, concrete, and pavement), with the highest cover at PL (79.5%) and the lowest at ML (21.1%).

### Eddy covariance measurements and data processing

The mobile EC platform consisted of a telescoping tower that extends to a maximum height of 15 m. High frequency measurements of *FC*, sensible heat (*H*), and latent heat *(λET*) fluxes were made using an infrared gas analyzer (LI-7500, Li-Cor Biosciences) to measure H_2_O and CO_2_ concentrations, and a three-dimensional sonic anemometer (CSAT3, Campbell Scientific) to measure wind velocities [58]. Sensors were aligned to the dominant wind direction for each deployment, which were determined as 21° at XL, 227° at PL, 230° at ML, and 259° at REF. EC measurements were carried out at a height of 7.0 (XL), 9.0 (PL), and 8.0 m (ML) to ensure that turbulent fluxes were observed above the urban roughness sublayer. In almost all the cases, the EC measurements were above the surrounding roughness elements within the footprint [56]. The average urban canopy layer height (*z*_*h*_) was 3.5, 2.8, and 5 m for the XL, PL, and ML sites, respectively, leading to estimated blending heights (1.5*z*_*h*_) from [47] which were smaller than measurement heights. Thus, we assume that the mobile measurements sampled a blended, spatially-averaged signal considered as representative of the urban land cover within the small footprint [68]. As a result, the application of Monin-Obukhov Similarity Theory and the concept of stability are valid [69]. The REF site had a taller height of 22.1 m, measuring turbulent fluxes from a broader and more heterogeneous residential area [7].

Data was collected at 10 (PL, ML, and REF) and 20 Hz (XL) and processed at 30-minute intervals using EdiRe [70]. EC processing included corrections for stability and density fluctuations [69, 70, 71], coordinate rotation [72], removal of signal lags in gas concentrations due to the separation between the sensors [73], frequency response corrections [74], and estimates of sensible heat using the sonic temperature corrected with humidity following standard procedures [75]. Measurements were also filtered to exclude periods when precipitation was > 0.2 mm per 30 min, when winds were from the opposite direction at which instruments were mounted, when fluxes were further than 3 standard deviations from the mean, when the friction velocity criterion of u* < 0.15 m s^-1^ was met, and for absolute values of *FC* greater than 2 mg m^-2^ s^-1^, according to the behavior of the 30-min values and to *Schmid et al*. [76]. Missing data due to data filtering and sensor malfunction accounted for 54.1%, 29.9%, 50.2% and 37.2% of the total half-hourly data obtained during the deployments at the XL, PL, ML, and REF sites, respectively. Most of the missing data corresponded to night-time measurements (80.5%, 70.2%, 59.6% and 74.3% at XL, PL, ML, and REF). Gap-filling procedures were not used to avoid the impacts that these methods might have on comparisons of daily values. Additional measurements at all sites included net radiation (*R*_*n*_) using a four-component net radiometer (CNR4, Vaisala), air temperature (*T*_*a*_), and relative humidity (*RH*) using a HMP155A probe (Vaisala), and precipitation (*P*) using a tipping-bucket rain gauge (TE525MM, Texas Electronics).

### Urban carbon dioxide budget and meteorological conditions

The urban CO_2_ budget varies from natural ecosystems due to anthropogenic sources. Urban *FC* is composed of sources, sinks, and storage changes [10, 68, 77] as:

$$FC=F_{F}+F_{R}-F_{P}+\Delta S_{C},$$ (1)

where *F*_*F*_ is the CO_2_ emitted from fuel combustion; *F*_*R*_ is the release of CO_2_ due to respiration by animals, humans, and vegetation; *F*_*P*_ is the CO_2_ assimilated by the photosynthesis of vegetation; and Δ*S*_*C*_ is the net changes of CO_2_ storage, generally considered to be small or negligible during fully turbulent conditions [78]. Consideration of the storage changes in the urban CO_2_ budget is relatively rare [32, 78]. Typically, *FC* is reported in grams (g) or milligrams (mg) of CO_2_ per unit area per unit time, while carbon dioxide concentrations ([CO_2_]) in the atmosphere are reported in parts per million (ppm). In practice, *FC* measurements in urban areas using the EC method are not able to identify the various origins of the CO_2_ fluxes. Nevertheless, a *FC* < 0 indicates that plant uptake is larger than respiration and anthropogenic emissions (*F*_*P*_ > *F*_*F*_ + *F*_*R*_, or a net carbon dioxide sink), while a positive *FC* suggests a net carbon dioxide source (*F*_*F*_ + *F*_*R*_ > *F*_*P*_). Neutral flux conditions (*FC* ≅ 0) occur when sources and sinks are balanced (*F*_*F*_ + *F*_*R*_ = *F*_*P*_).

*FC* and the associated meteorological conditions for each sampling period at each site were analyzed at various time scales: (1) daily averages, (2) average diurnal cycles at 30-min resolution, and (3) total amounts during the sampling period. From the large set of measurements, we focus on *P*, *T*_*a*_, *RH*, and incoming solar radiation (*R*_*s*_). For the EC systems, *R*_*n*_ is obtained from measurements of the net shortwave (*R*_*s*_^*net*^) and net longwave (*R*_*l*_^*net*^) radiation as:

$$R_{n}=R_{s}^{net}+R_{l}^{net}=\left(1-a\right)R_{s}+R_{l}^{net},$$ (2)

where *a* is the albedo, with all radiation fluxes measured in W m^-2^. As described in *Templeton et al*. [58], the surface energy balance for a simple plane facet in an urban area, under the assumptions of negligible anthropogenic heat, advection and energy storage, can be described as:

$$R_{n}-G=H+\lambda ET,$$ (3)

where *G* is the ground heat flux, *H* is the sensible heat flux, and *λET* is the latent heat flux, all in W m^-2^. Evapotranspiration (*ET* in mm day^-1^), obtained using the latent heat of vaporization (*λ*), is analyzed at daily and diurnal time scales. Furthermore, we estimated the evaporative fraction (*EF*) as a daily average and for the daytime period (at 30-min resolution) as:

$$EF=\frac{\lambda ET}{H+\lambda ET},$$ (4)

to provide insight into the relation between *FC* and the turbulent fluxes. Additional analyses were performed for subsets of days classified as ‘wet’ or ‘dry’ based on the occurrence of precipitation (*P* > 0.2 mm day^-1^) on the day of an event and the two subsequent days.

### Analyses of controlling factors with ancillary data

We related the *FC* measurements to anthropogenic and biogenic processes that lead to sources and sinks of CO_2_ in urban environments. According to *Koerner and Klopatek* [20], around 80% of the total CO_2_ contribution in the PMA is due to vehicular traffic. As such, we analyzed *FC* separately for weekdays (Monday to Friday) and weekends (Saturday and Sunday) and related these to vehicular traffic counts for nearby streets to the deployments as well as to the areal fraction of pavement classified for each site. Traffic counts (total of vehicles, 2-way hourly resolution data) were obtained through the Traffic Counts Database System of the Maricopa Association of Governments (http://mag.ms2soft.com/tcds/tsearch.asp?loc=Mag&mod=). Since traffic counts data was limited, we obtained the available information for dates close to the sampling periods (3/18/2015 to 3/24/2015 for XL, 2/25/2015 and 2/26/2015 for PL, 2/18/2015 to 2/24/2018, and 3/4/2015, 3/5/2015, 3/24/2015 and 3/25/2015 for REF) and for streets near to the deployments. While biogenic factors in urban environments include both vegetation and soil activity, plants are the only known sink of CO_2_ that can oppose anthropogenic emissions [12]. Thus, we analyzed the effect of vegetation activity through the fraction of trees and grasses at each site as well as the Normalized Difference Vegetation Index (NDVI) obtained from the Moderate Resolution Imaging Spectroradiometer (MODIS) product MOD09GQ MODIS/Terra Surface Reflectance Daily L2G Global 250m SIN GRID [79]. Data obtained corresponded to the 250 m pixel at each site for each sampling period. The complete dataset included NDVI images from January 1 to October 13, 2015, with cloud-free scenes (190 out of 286). However, the coarse resolution of MODIS presents a scale discrepancy to the source areas around each tower. To compensate for this, NDVI from the higher resolution Landsat 8 OTI product was used to bias-correct the MODIS data through a linear regression over each site. A comparison of *FC* was also conducted separately for sunny and cloudy days using a threshold of measured *R*_*s*_ (75% of the seasonal daily average). To determine if the *FC* observations were different between selected days (i.e., weekday versus weekend or sunny versus cloudy), a Mann-Whitney U Statistic rank sum test was performed, with significance determined for *p* ≤ 0.05. A similar procedure was conducted to quantify the effect of wet versus dry conditions on the *FC*, [CO_2_], and *EF* observations. Pearson correlation coefficients (CC) were also used to test the linear correlation (-1 ≤ CC ≤ 1) between measured variables, among different sites and with controlling factors such as NDVI, with significance determined for *p* ≤ 0.05. For all statistical analyses, only daytime data were used to focus on time periods when the controlling factors affect the measured fluxes.

## Results and discussion

### Seasonal variations in meteorological and CO_2_ conditions

**Fig 2:**
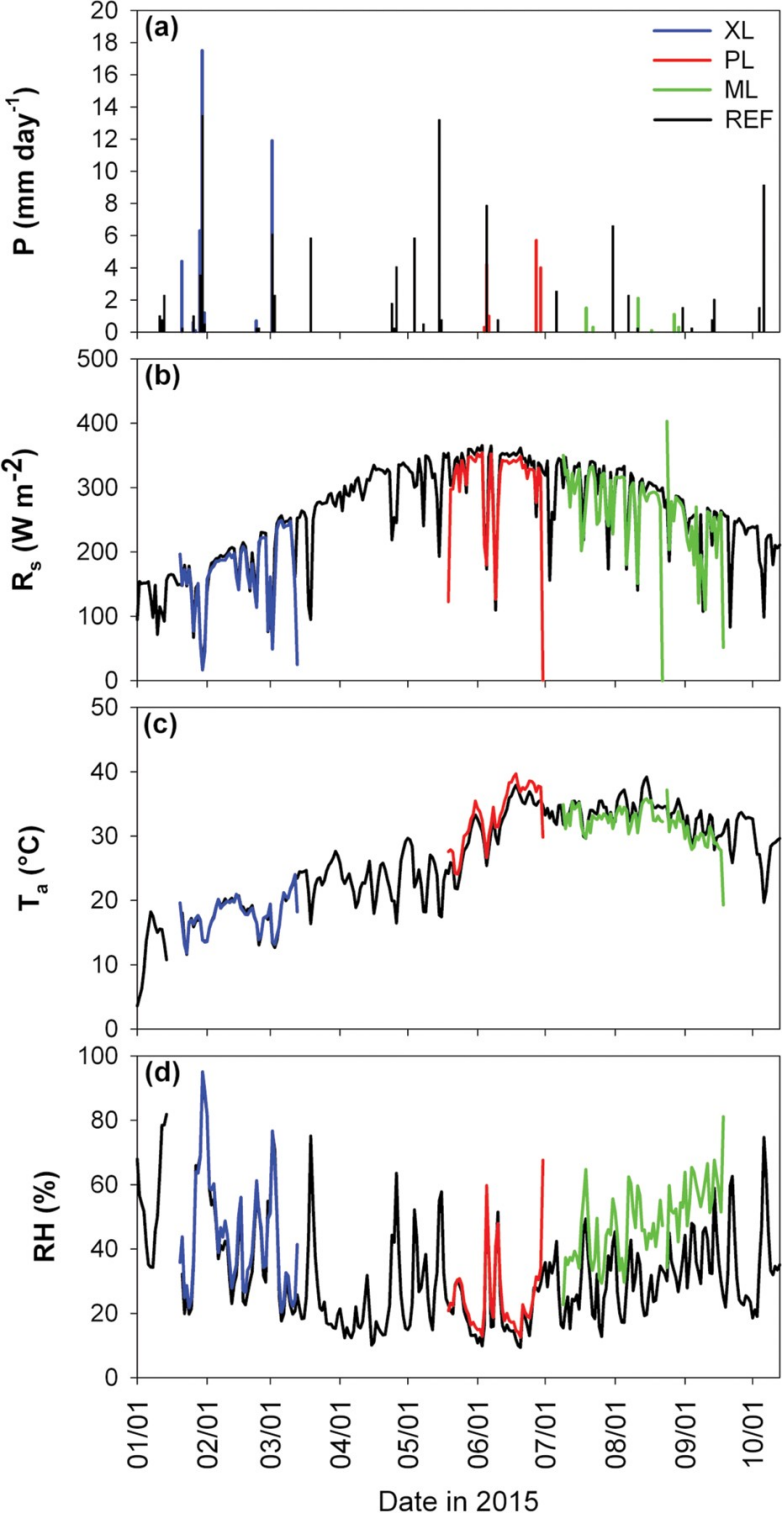
Meteorological conditions in the four sites. Comparison of meteorological measurements during entire study period (1 January to 30 September, 2015) including: (a) precipitation, (b) incoming solar radiation, (c) air temperature, and (d) relative humidity, shown as daily averages (*R*_*s*_, *T*_*a*_, and *RH*) or totals (*P*).

**Fig 3:**
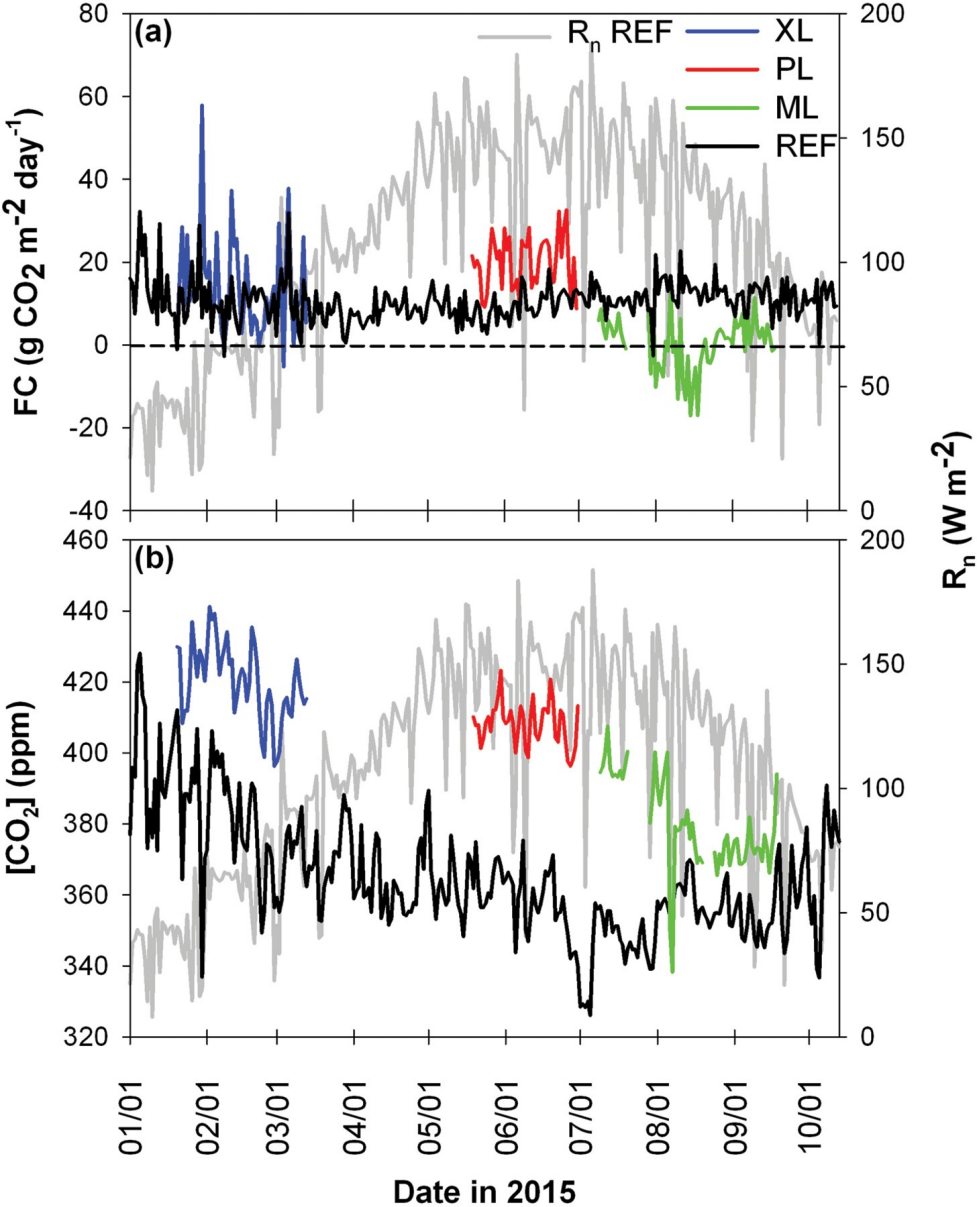
Daily values of *FC* for each site. Daily total values of *FC* (a) and average values of CO_2_ concentration (b) for the XL, PL, ML, and REF sites over the study period, with daily average values of net radiation (*R*_*n*_) at REF shown as a reference.

Daily values of precipitation (total in mm), incoming solar radiation (average in W m^-2^), air temperature (average in °C) and relative humidity (average in %) are shown in Fig 2 for the three deployments and the REF site. Temporal variations in meteorological variables reflect the seasonal progression from winter to summer and the influence of individual precipitation events occurring across all seasons. To complement this comparison, Table 3 presents differences in *P* and *T*_*a*_ between the mobile deployments and reference site during simultaneous periods. Note that the sampling periods were generally drier and warmer than corresponding long-term (1981–2010) averages [58]. Overall, the XL site had similar meteorological conditions as the REF site during the same period with a small difference in *R*_*s*_ (average of -6.57 W m^-2^ lower at XL), due to the higher *P* during the winter-spring period. Similarly, the PL site had lower values of *R*_*s*_ (-11.64 W m^-2^), but higher *T*_*a*_ (+1.90°C) and similar *RH*, as compared to the REF site at the daily scale. At the ML site, a lower *R*_*s*_ (-12.98 W m^-2^) and *T*_*a*_ (-1.83°C) were measured, with an increased amount of *RH* (+15.83%) due to the frequent irrigation of the turf grass. Noted small differences in meteorological conditions are due to a number of factors: (1) small variations in the sensor types and deployment heights [7, 58], (2) daily differences in precipitation and cloud cover at sites which were at most 42.8 km apart (Figs 1 and 3) the effects of land cover on surface properties, including albedo, soil temperature and soil moisture, that influence meteorological states through the surface energy balance, as discussed in *Templeton et al*. [58].

**Table 3:** Total *FC*, average [CO_2_], precipitation and air temperature during each deployment and for the simultaneous period at the REF site (labeled as Ref.).

Site	*FC* (g CO_2_ m^-2^)	Ref. *FC* (g CO_2_ m^-2^)	[CO_2_] (ppm)	Ref. [CO_2_] (ppm)	*P* (Ref. *P*) (mm)	*T*_*a*_ (Ref. *T*_*a*_) (°C)
**XL**	723.31	561.54	419.89	379.44	43.0 (27.7)	17.3 (17.8)
**PL**	862.40	406.13	408.05	358.95	15.2 (8.6)	32.9 (31.6)
**ML**	-166.30	769.26	380.24	353.49	5.4 (13.7)	33.1 (33.5)
**REF**	3021.56	3021.56	364.84	364.84	99.6 (99.6)	26.5 (26.5)

Fig 3 presents the seasonal variation of daily values of *FC* (total in g CO_2_ m^-2^ day^-1^) and [CO_2_] (average in ppm) for the three mobile deployments in comparison to the REF site. Daily averages of net radiation (*R*_*n*_) are shown to distinguish seasonality. Clear differences are noted in the magnitude and behavior of *FC* and [CO_2_] between the sites, as quantified in Table 3. XL, PL, and REF acted as net sources of carbon dioxide during the sampling period, while ML was a carbon dioxide sink. All mobile sites had higher [CO_2_] than the REF site, while a larger (smaller) *FC* was noted at XL and PL (at ML) when compared to REF. Lower values of [CO_2_] were measured at the REF site due to a much higher sampling height than the mobile deployments since [CO_2_] decreases with altitude. As expected, [CO_2_] decreases from winter to summer in response to seasonal variations in northern hemisphere vegetation activity [80]. Except for the early part of the year, the REF site exhibits a fairly constant *FC* during the period (average of 10.56 g CO_2_ m^-2^ day^-1^) and a narrow range of fluctuations (standard deviation of 4.82 g CO_2_ m^-2^ day^-1^). This is within the ranges of values (in g CO_2_ m^-2^ day^-1^) for other open low-rise sites, for instance, in Melbourne, Australia from 8.49 to 33.4 [37] and in Syracuse, USA with 11.23 [13]. In contrast, the XL site had wide variations in daily *FC* (std. of 11.39 g CO_2_ m^-2^ day^-1^) with magnitudes (ave. of 13.64 g CO_2_ m^-2^ day^-1^) that were generally higher than at REF as well as higher [CO_2_] (+44.25 ppm). This value is similar to an open mid-rise site measured in Sakai, Japan with 12.8 [15], but lower than year-round values reported in Tokyo, Japan [48], Mexico City [28], and Essen, Germany [24], at 43, 35.4 and 35.4 g CO_2_ m^-2^ day^-1^. Daily fluctuations at XL correspond to changes in vehicular traffic and plant phenology.

The PL site had consistently higher *FC* values as compared to the REF site (ave. 20.05 g CO_2_ m^-2^ day^-1^), with larger daily variations (std. 6.39 g CO_2_ m^-2^ day^-1^) and higher [CO_2_] (+49.38 ppm). The only previous study with a large low-rise structure was found in Houston, USA [81], which reported a higher daily value (29.38 g CO_2_ m^-2^ day^-1^) during the summer, however, other highly urbanized sites including compact low-rise [13, 38, 39, 53], compact mid-rise [12, 24, 48, 59, 82–86] and compact high-rise [50], reported values between 18.7 and 71.7 g CO_2_ m^-2^ day^-1^ during the summer. The Pearson correlation coefficient (CC) of *FC* between XL and PL and REF was significant (CC = 0.31 and 0.37, respectively), whereas ML had a much lower, insignificant correlation (CC = -0.15), suggesting a stronger similarity in the factors affecting *FC* at these two sites (see Table 4 for a comparison of CC for other variables between the mobile deployments and REF site). A distinct behavior is noted at the ML site, where a more negative *FC* (ave. -2.77 g CO_2_ m^-2^ day^-1^) is observed as compared to the REF site, with similar daily variations (std. 7.65 g CO_2_ m^-2^ day^-1^), while a slightly higher [CO_2_] (+25.91 ppm) is due to the lower sampling heights. Gradual variations during the summer could correspond to vegetation uptake, with a minimum value of ≈ -17 g CO_2_ m^-2^ day^-1^ in August and positive values at the end of the sampling period. Sparsely built sites in other cities had also daily values close to zero showing that vegetation can neutralize CO_2_ emissions for a particular season, for instance, during the summer in Copenhagen, Denmark [59], Saint Paul, USA [87], and Montreal, Canada [30]. Other highly vegetated urban sites showed CO_2_ uptake values during the summer, for example in Baltimore, USA [32] and in Nagoya, Japan [88].

**Table 4:** Pearson correlation coefficient (CC) of daily values of precipitation, air temperature, net radiation, latent heat flux, evaporative fraction, carbon dioxide flux, and carbon dioxide concentration between mobile locations and REF site for simultaneous periods.

Site	*P*	*T*_*a*_	*R*_*n*_	*λET*	*EF*	*FC*	[CO_2_]
**XL**	**0.95**	**0.95**	**0.88**	**0.62**	**0.84**	**0.31**	**0.69**
**PL**	**0.48**	**0.96**	**0.84**	**0.48**	**0.38**	**0.37**	**0.69**
**ML**	-0.02	**0.76**	**0.68**	**0.34**	0.14	-0.15	-0.08

Bolded numbers indicate significant correlations at *p* ≤ 0.05.

### Diurnal variations in surface energy, water and CO_2_ conditions

**Fig 4:**
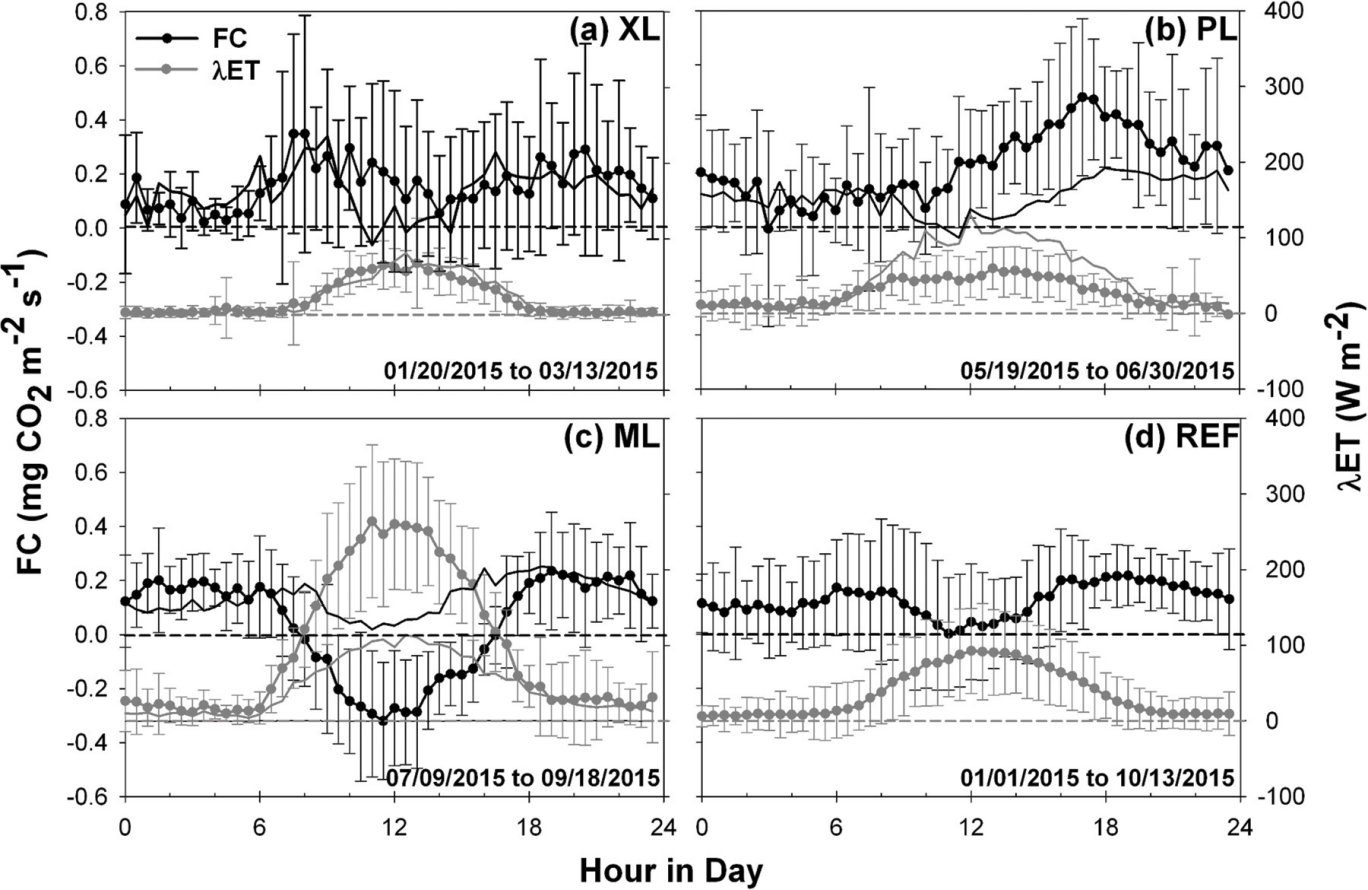
Diurnal averages of *FC* and *λET* at the four sites. Average diurnal cycle of carbon dioxide flux and latent heat flux for (a) XL, (b) PL, (c) ML, and (d) REF sites. Error bars represent one standard deviation over the indicated periods. Thin lines are average diurnal cycles at the REF site during the same period as the mobile deployment for *FC* (black) and *λET* (gray). Horizontal lines indicate zero values. Different sampling periods are specified in each plot.

Diurnal variations of carbon dioxide flux and latent heat flux are compared in Fig 4, where symbols indicate average values at 30-min resolution and error bars capture ±1 standard deviation during each deployment period (i.e., for differing seasons). For comparison, thin lines show corresponding values at the REF site for the same periods as the deployments at XL, PL, and ML. Clear variations are noted in *FC* and *λET* among the sites. For instance, latent heat flux, which can be a proxy for irrigated vegetation activity due to its dependence on well-watered plant transpiration [89], varies considerably [58], with average daily peaks of 67.82 W m^-2^ (XL), 59.54 W m^-2^ (PL), 263.96 W m^-2^ (ML), and 92.48 W m^-2^ (REF). The largest diurnal peaks of *λET* correspond to ML (Fig 4C) that is composed of the highest fraction of irrigated vegetation (44.3%) and exhibits the most negative values of *FC* near mid-day (-0.32 mg CO_2_ m^-2^ s^-1^). The diurnal behavior of *FC* and *λET* at ML is similar to observations in natural ecosystems during well-watered conditions or neighborhoods with abundant vegetation [10, 13, 15, 22, 30, 39, 55, 87, 88, 90, 91]. Furthermore, the difference in time between the diurnal minimum in *FC* and the maximum in *λET* is short, about 0.5 hours on average, showing a coupling between *FC* and *λET* that is typical of natural ecosystems where maximum photosynthesis occurs near mid-day.

In contrast, the PL site (Fig 4B) has a reduced amount of *λET* with the least variation during the day due to its low fraction of vegetation (6.6%), resulting in a positive *FC* during the day, with a peak of +0.48 mg CO_2_ m^-2^ s^-1^ at 5:00 p.m. coinciding with rush hour traffic in the nearby street. This leads to a decoupling of the peaks in *FC* and *λET*, which are separated by 4 hours at PL. Peaks of *FC* during rush hours are typical of highly urbanized areas, with reported values between 0.35 to 1.67 mg CO_2_ m^-2^ s^-1^ in compact low-rise and mid-rise areas during the summer [12–15, 82, 92] and about 0.62 mg CO_2_ m^-2^ s^-1^ in a compact high-rise during the summer [50]. Interestingly, the diurnal cycles at XL and REF (Fig 4A and 4D) exhibit behaviors that are a mixture of the effects of vegetation and traffic activity. Positive peaks in *FC* occur around rush hour times of 8:00 a.m. and 6:00 p.m. (0.35 and 0.26 mg CO_2_ m^-2^ s^-1^ at XL; 0.16 and 0.21 mg CO_2_ m^-2^ s^-1^ at REF, respectively), while a mid-day minimum in *FC* is noticeable (0.05 and 0.02 mg CO_2_ m^-2^ s^-1^ at XL and REF, respectively). The decreases in *FC* coincide with vegetation activity due to the small differences in time with *λET* (±1.5 hours), but are insufficient to counteract CO_2_ emissions, such that *FC* remains positive at XL and REF on average during the course of a day. This behavior have been reported in several open low- and mid-rise urban landscapes, with summer values fluctuating from 0.13 to 1.32 mg CO_2_ m^-2^ s^-1^ during rush hours and mid-day values from ~0 to 0.44 mg CO_2_ m^-2^ s^-1^ [13, 15, 16, 24, 28, 37, 47, 48, 92, 93].

**Fig 5:**
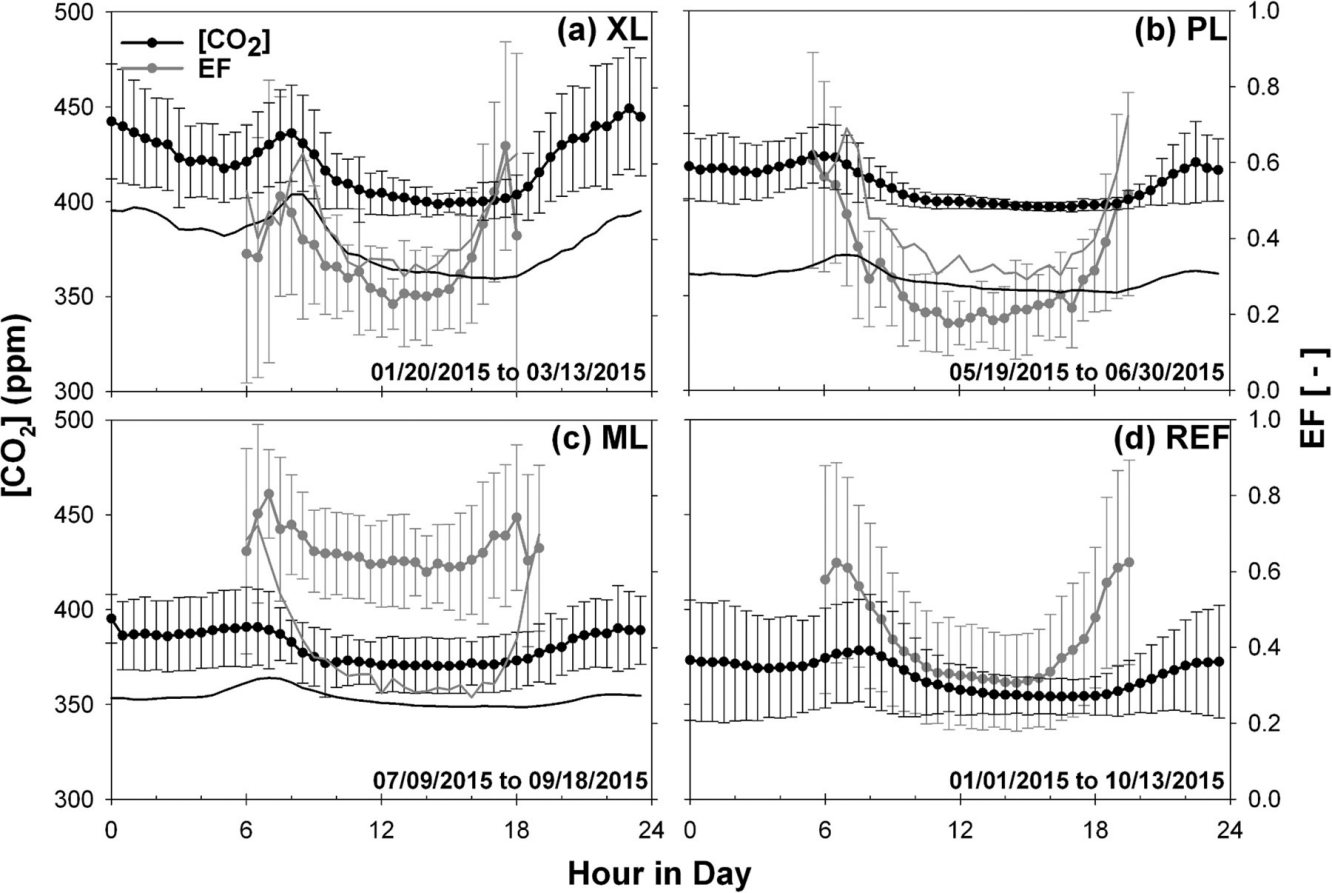
Diurnal averages of [CO_2_] and *EF* for the four sites. Average diurnal cycle of CO_2_ concentration and evaporative fraction for the (a) XL, (b) PL, (c) ML, and (d) REF sites. Error bars represent one standard deviation over the indicated periods. Thin lines are average diurnal cycles at the REF site during the same period as the mobile deployment for [CO_2_] (black) and *EF* (gray). Different sampling periods are specified in each plot.

To complement this analysis, Fig 5 presents the diurnal cycles of CO_2_ concentration and daytime evaporative fraction during each deployment period. Given the stronger variation in [CO_2_] from winter to summer relative to *FC* (Fig 3), it is useful to directly compare the mobile deployments to the simultaneous behavior at the REF site (thin lines). Relatively small variations in [CO_2_] occur throughout the day, with standard deviations of 9.45 ppm (XL), 17.11 ppm (PL), 8.46 ppm (ML), and 8.15 ppm (REF). Higher [CO_2_] typically corresponds to morning traffic periods from 6:00 to 8:00 a.m. and in the evening from 6:00 to 10:00 p.m. when the diurnal accumulation of CO_2_ and ceasing of plant uptake play a role as well as changes in the urban boundary layer height [94]. Relative to the REF site, PL has the highest [CO_2_] and exhibits the strongest diurnal variations, in part due to its high fraction of urban surfaces dedicated to transportation (79.5%, Fig 1D) including the parking lot and nearby streets, particularly during the afternoon and night due to the nature of the surrounding businesses. During mid-day, pavements and buildings at PL have the lowest *EF* (0.18), an indication that surface energy fluxes are dominated by conduction from urban materials. In contrast, irrigated turf grass and trees at the ML site support a much higher mid-day *EF* (0.61), whose daytime variations match well with the observed decrease in [CO_2_] in response to plant uptake. Notably, the higher overall magnitude of [CO_2_] at ML relative to REF (Table 3) is likely due to differences in sampling height (8 m versus 22.1 m) as the decrease in [CO_2_] with altitude is well known [23, 95]. This is supported by the higher daytime *EF* at ML during a simultaneous period comparison with REF (thin line) which is consistent with more negative *FC* at the lower sampling height of ML (Fig 4C). In between these end-member cases, XL and REF exhibit diurnal behaviors with respect to [CO_2_] and *EF* that are mixtures of plant uptake and vehicular emissions. Thus, to isolate the effects of these factors requires a more detailed view of site conditions, as described next. Additionally, [CO_2_] dynamics are affected by diurnal changes in boundary layer conditions such as vertical mixing and advection [83, 94], however, those factors are not analyzed here.

### Controlling factors of CO_2_ conditions

**Fig 6:**
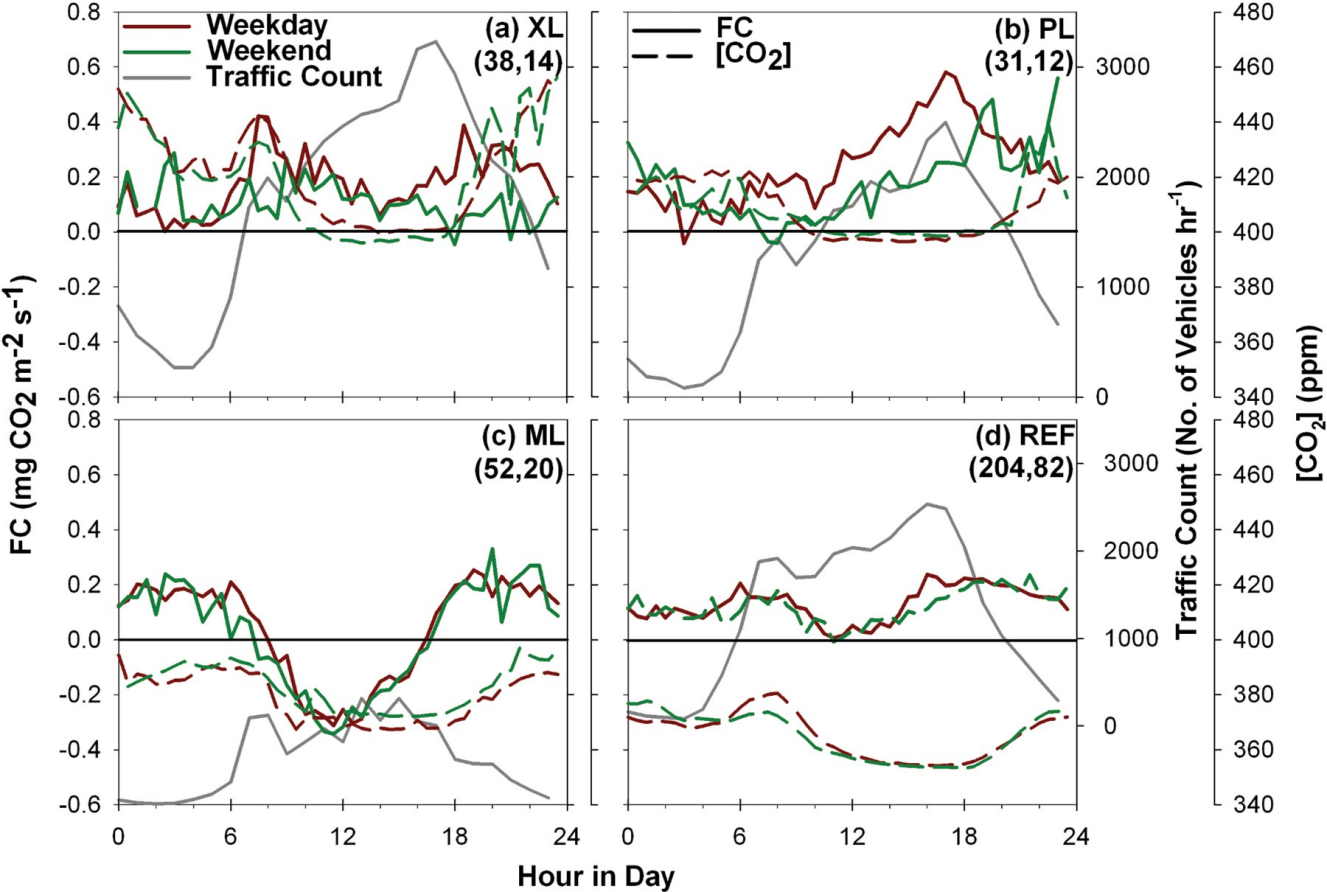
Diurnal averages of *FC* for weekday and weekend days. Comparison of the average diurnal cycles of carbon dioxide flux for weekday and weekend days for the (a) XL, (b) PL, (c) ML, and (d) REF sites, including traffic counts from nearby streets. Parentheses indicate the number of days in each category of weekday and weekend for each site, respectively. Different sampling periods are specified in each plot.

The effect of vehicular traffic is assessed in Fig 6 through comparisons of the average diurnal cycle of *FC* and [CO_2_] for weekday and weekend days at each site. This is an approach that has been used in several studies to assess the impacts of traffic on *FC* [13, 16, 28, 30, 34, 37, 38, 39, 82, 86, 93, 96]. For reference, local traffic counts (number of vehicles per hour) are provided as diurnal cycles for available time periods. Differences in *FC* between weekday and weekend periods are noted for rush hour periods (8:00 a.m. and 6:00 p.m.) at the XL, PL, and REF sites, coinciding with higher traffic counts. Similarly, [CO_2_] exhibits higher values at these sites for weekdays when a higher traffic volume is expected, but typically only in the morning. Larger *FC* and [CO_2_] differences at PL (ave. of 0.18 mg CO_2_ m^-2^ s^-1^ and 3.57 ppm) between weekday and weekend days suggest that the CO_2_ budget in the parking lot is controlled primarily by vehicular emissions in nearby streets. In addition, a progressive increase in *FC* is noted at PL during the daytime hours for all days, closely matching the rise in traffic. In contrast, all other sites are characterized by a mid-day decrease in *FC* and [CO_2_], despite rising traffic counts at XL and REF, which is attributed to vegetation uptake counteracting the vehicular emissions for *FC* and an increase of the urban boundary layer height during the day for [CO_2_] [84]. Smaller differences in *FC* and [CO_2_] at XL and REF (ave. of 0.03 and 0.01 mg CO_2_ m^-2^ s^-1^ and 5.88 and 2.69 ppm, respectively) between weekday and weekend days, as well as the mid-day response to plant activity, suggest that these sites are influenced by both traffic and vegetation factors for the sampled season at XL and over the entire period for REF. At ML, however, the amount of vegetation activity overwhelms the possible influence of traffic on daytime *FC* and [CO_2_]. Negligible differences (ave. of 0.01 mg CO_2_ m^-2^ s^-1^ and -2.88 ppm) are noted between weekday and weekend days, suggesting the CO_2_ budget in the well-irrigated mesic landscaping is controlled by photosynthetic uptake of CO_2_ by turf grass and trees. A decrease in *FC* during weekends has also been found for open low-rise areas [13, 16, 30, 37, 93], open and compact mid-rise sites [28, 39, 82, 86, 96], compact low-rise locations [13, 38, 29] and some sparsely built areas [30]. In contrast, this effect has not been noted in highly-vegetated urban areas [34].

**Fig 7:**
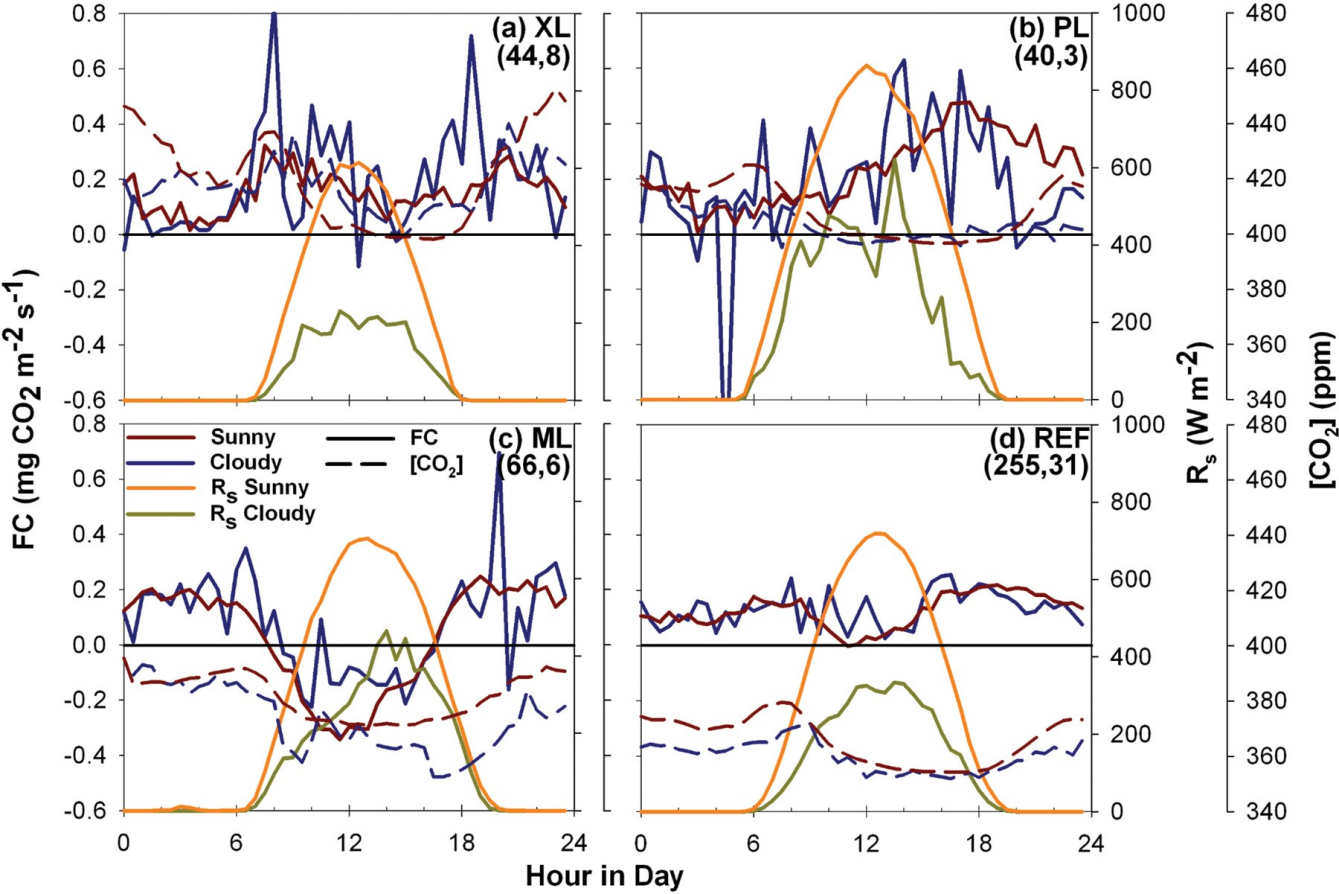
Diurnal averages of *FC* for sunny and cloudy days. Comparison of the average diurnal cycles of carbon dioxide flux for sunny and cloudy days for the (a) XL, (b) PL, (c) ML, and (d) REF sites, including average diurnal cycles of incoming solar radiation. Parentheses indicate the number of days in each category of sunny and cloudy for each site, respectively. Different sampling periods are specified in each plot.

To isolate the vegetation controls, Fig 7 compares the average diurnal cycle of *FC* and [CO_2_] for sunny and cloudy days during both weekday and weekend days with the incoming solar radiation for each category shown as a reference. This analysis allows inspecting the effect of plants on the CO_2_ budget as sunny (cloudy) days promote (diminish) photosynthetic activity, whereas the value of *R*_*s*_ should not impact other controlling factors such as traffic. Prior work has compared *FC* with radiation data or plant phenology to analyze the role of vegetation on urban carbon dioxide fluxes [10, 13, 15, 30, 55]. Since less than 20% of the days in a year are cloudy in the PMA [97], we use the effects of clouds on radiation to discern the role of photosynthesis. During the study period, the percentage of cloudy days were 15.4%, 7.0%, 8.3% and 10.8% of the sampling durations at XL, PL, ML, and REF, respectively. In addition, CC values between NDVI and CO_2_ conditions are shown in Table 5 as a means to determine if significant relationships exist with vegetation development. Sunny days generally lead to lower *FC*, but not necessarily to lower [CO_2_], in particular during mid-day, with average differences of -0.08 (XL), 0.0 (PL), -0.07 (ML), and -0.02 mg CO_2_ m^-2^ s^-1^ (REF); and -3.61 (XL), 3.16 (PL), 9.98 (ML), and 3.89 ppm (REF), respectively. *FC* response is consistent with the vegetation fractions at XL (38.6%), PL (6.6%), ML (44.3%), and REF (14.6%). In addition, cloudy days exhibit higher *FC* during rush hour periods, suggesting that low vegetation activity in these few days in the PMA (note the low number of cloudy days) cannot counteract vehicle emissions, except at ML, where a significant change is not shown. Statistically significant relations with daily NDVI are only noted with [CO_2_] at the REF site, suggesting that plant development has a minimal impact on *FC* or [CO_2_] over the progression of each period. While there was plant development and phenological features observed during the study periods, these do not significantly impact the CO_2_ budget during the deployments of XL, PL and REF but have an impact at ML. Furthermore, despite the mid-day decrease, vegetation activity did not completely counteract *FC* in XL, PL and REF, as these locations acted as net sources of carbon dioxide, however, vegetation activity in ML was enough to counteract this effect. These results are consistent with those reported in other cities, where highly-urbanized areas are insensitive to changes in radiation, but highly-vegetated landscapes show differences between days with high or low radiation [10, 15, 30, 55].

**Table 5:** Pearson correlation coefficient (CC) of daily values of normalized difference vegetation index with net radiation, latent heat flux, evaporative fraction, carbon dioxide flux and carbon dioxide concentration at all sites for simultaneous periods.

Site	*R*_*n*_	*λET*	*EF*	*FC*	[CO_2_]
**XL**	0.00	0.13	0.24	-0.03	-0.17
**PL**	-0.08	0.03	-0.08	-0.25	-0.05
**ML**	0.07	0.39	0.09	0.04	-0.08
**REF**	**-0.21**	**-0.25**	-**0.23**	**-0.17**	**0.24**

Bolded numbers indicate significant correlations at *p* ≤ 0.05.

**Fig 8:**
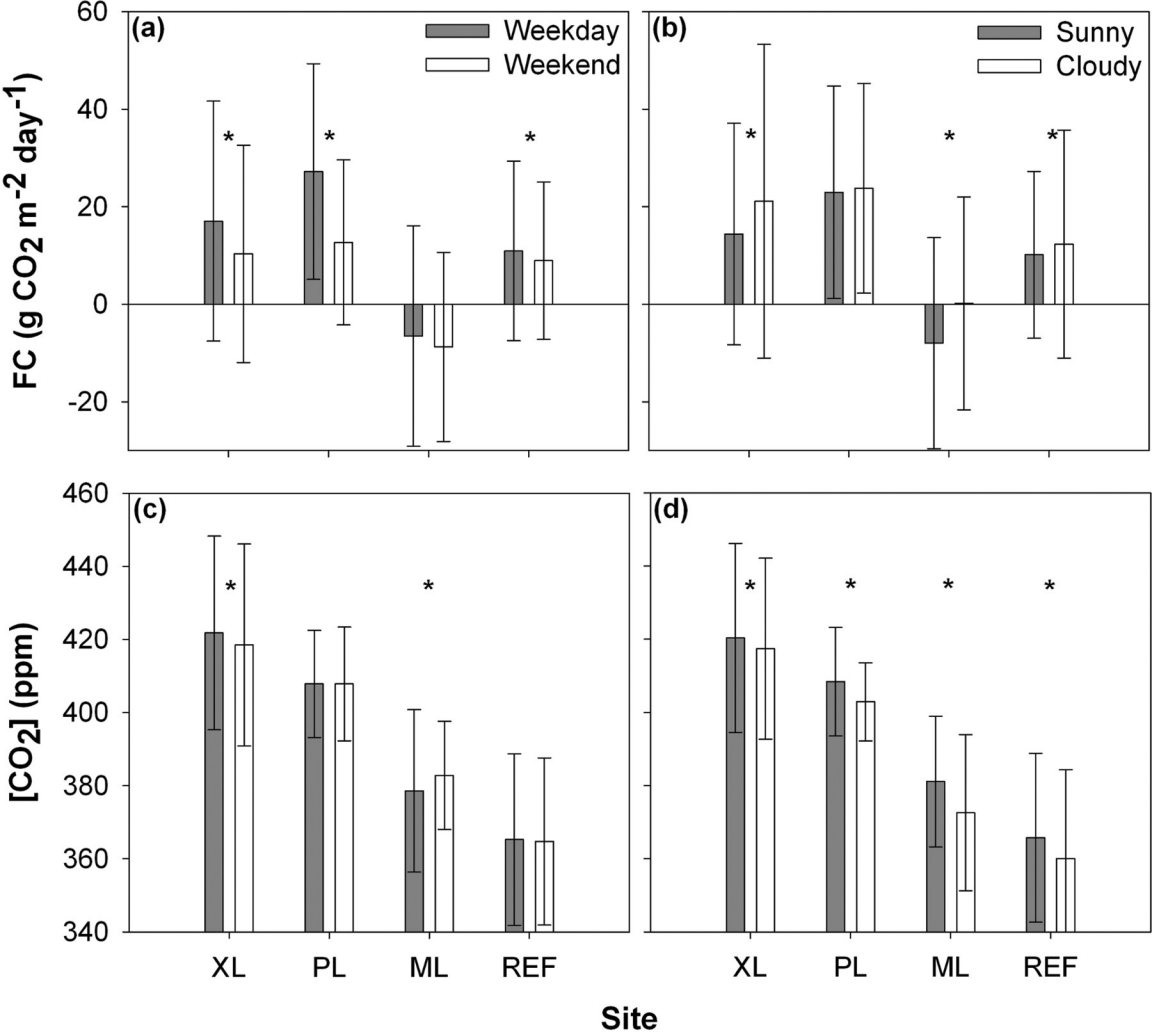
Average comparison between weekdays and weekends and sunny and cloudy days. Average values (bars) and standard deviations (error bars) of CO_2_ flux and concentrations for (a, c) weekday and weekend, and (b, d) sunny and cloudy days for the XL, PL, ML, and REF sites. Stars indicate significant differences within each site (*p* ≤ 0.05).

To summarize the controls on CO_2_ conditions, Fig 8 presents the average *FC* and [CO_2_] over each deployment period (and standard deviations as error bars) for weekday and weekend days (traffic effect), and for sunny and cloudy days (vegetation effect), including an indication of statistically significant differences (*p* ≤ 0.05). As noted earlier, the XL and REF sites exhibit controls on *FC* that reflect a mixture of the effects of plant uptake and vehicular emissions, while a significant traffic effect is not present on [CO_2_] at the REF site. In contrast, the PL site is dominated by traffic effects with no statistically significant impact of vegetation activity on *FC*, though cloudy conditions impact [CO_2_] likely due to the effects of rainfall washout [98]. Finally, the *FC* at the ML site is determined by plant uptake effects with no significant impact of traffic, whereas both controlling factors play a role on [CO_2_]. The lower [CO_2_] for cloudy days could result from washout and local modifications by storm events [98]. These results are consistent with the distribution of urban land cover types at each site, in particular the fraction of transportation surfaces and irrigated plants.

### Sensitivity to precipitation and urban irrigation

**Fig 9:**
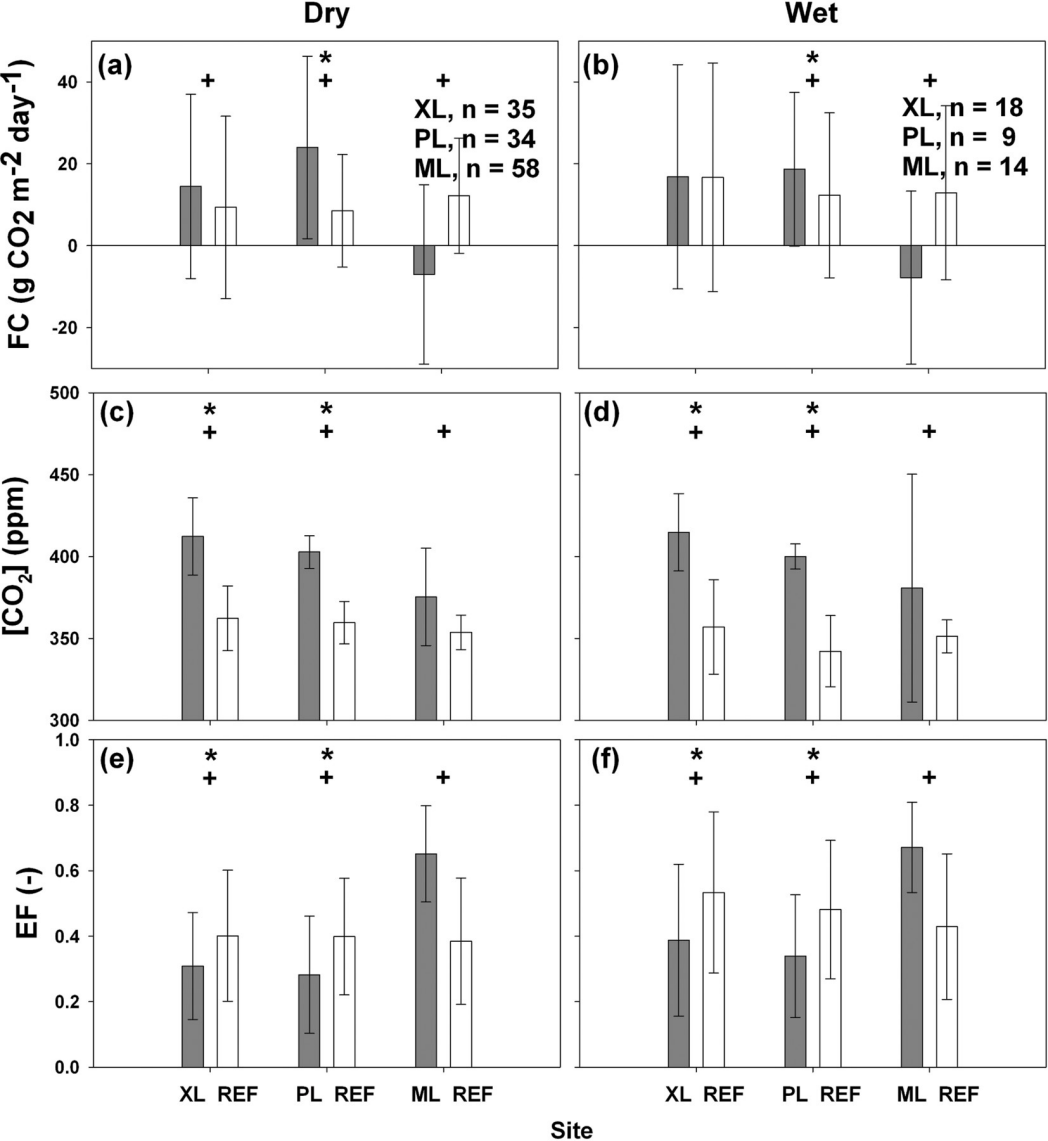
Daily averages of *FC*, [CO_2_] and *EF* for dry and wet days. Comparisons of averaged daily (a, b) *FC*, (c, d) [CO_2_], and (e, f) *EF* for dry (left) and wet (right) days during simultaneous periods. *n* is the number of days and error bars represent ±1 daily standard deviations. Statistical significance (*p* ≤ 0.05) is tested between dry and wet days in the same site (shown with *) and between the mobile deployment and REF site (shown with +).

The sensitivity of daily *FC*, [CO_2_], and *EF* to precipitation occurrence is assessed in Fig 9 through comparisons between wet and dry days at each study site. Wet days include those days with *P* > 0.2 mm day^-1^ and the two subsequent days after the precipitation event to account for moist soil conditions. Significance tests (*p* ≤ 0.05) are conducted between wet and dry days (labeled with *) within each deployment as well as between each mobile site and REF for simultaneous periods (labeled with +). While storm events are infrequent (note the lower *n* for wet days), these lead to significantly higher *EF* at the XL and PL sites with relatively lower amounts of vegetation, but no effect at the irrigated turf grass of the ML site. As discussed in *Templeton et al*. [58], this is likely due to the mesic or well-watered conditions at ML which maintain high *EF* that is insensitive to additional water input from storm events. Both wet and dry days have statistically significant differences in *EF* between each deployment and the REF site. CO_2_ concentrations vary significantly between wet and dry days at XL and PL, but not at the ML site, likely due to the negligible influence of precipitation on turf grass conditions. As expected, there are significant differences in [CO_2_] between each deployment and the REF site due to the effect of different sensor heights. Similarly, daily *FC* varies significantly between the mobile and REF sites for both dry and wet days, attributed to differences in CO_2_ emissions by vehicles and uptake by vegetation. However, the effect of precipitation occurrence was just evident at the PL site which had the lowest vegetation fraction. At the XL and ML sites, where a sufficient level of irrigated vegetation is present, *FC* does not significantly change in response to the additional water provided by storm events, though small increases in *FC* are present for wet days. The lower sensitivity of *FC* to rainfall at both the ML and XL sites suggests that plant photosynthesis occurs under well-watered conditions at these locations, whereas the use of *EF* as a diagnostic tool of this effect [58] identifies only the ML site as functioning as a mesic site.

**Fig 10:**
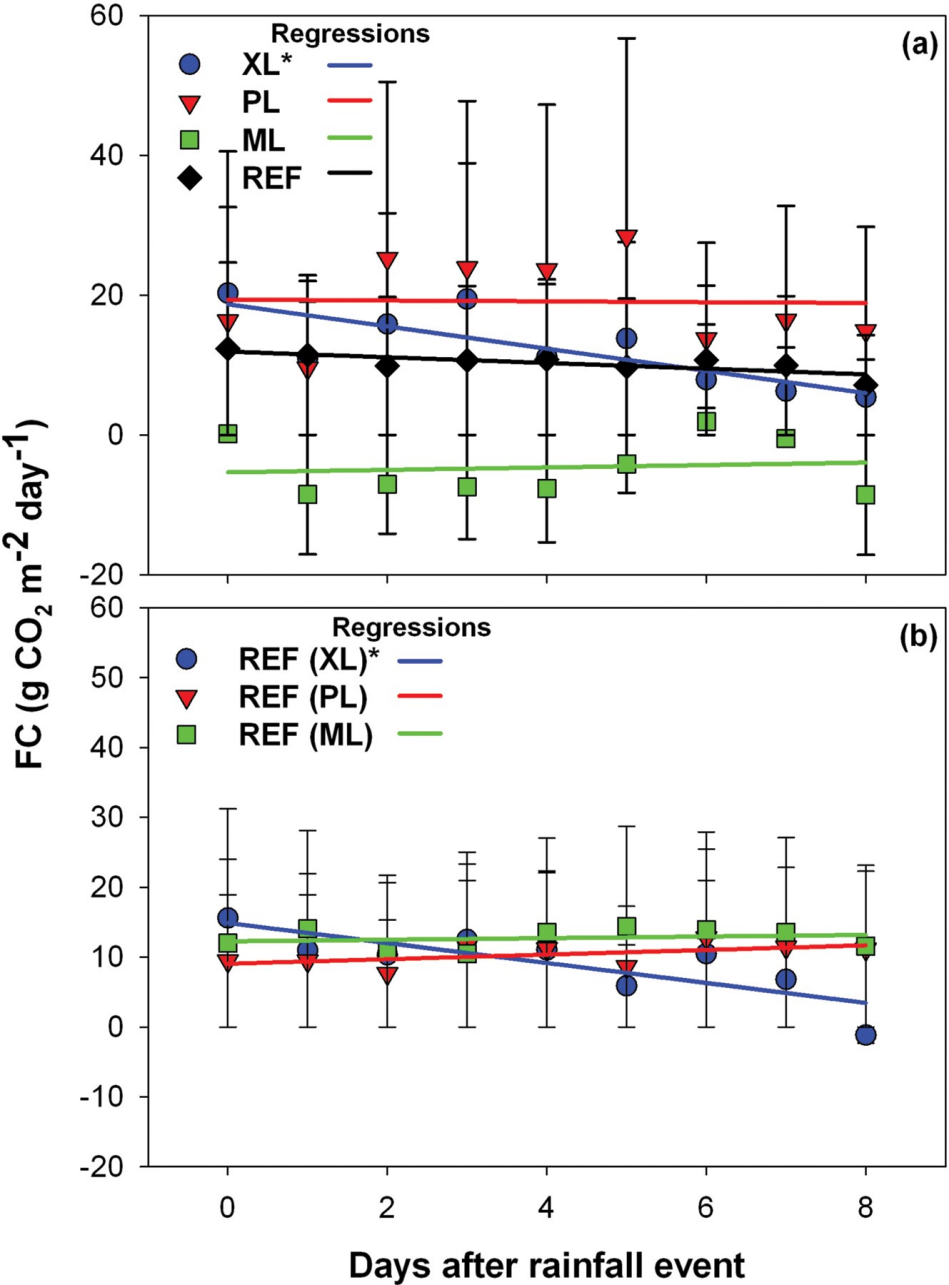
Response of daily *FC* to precipitation pulses. Daily *FC* as a function of days after a rainfall event for (a) all sites during their respective deployment periods, and (b) for the REF site during periods equal to temporal deployments at XL, PL, and ML. Symbols indicate averages and error bars depict ±1 standard deviation across all events. The linear regression slope is significantly different from zero at *p* ≤ 0.05 for the regressions labeled with an asterisk (*).

To explore this further, Fig 10 describes the response of *FC* to precipitation input for the sequence of days after rainfall at each study site. In this analysis, all periods after every storm are analyzed by inspecting the daily *FC* to obtain an average value for all events, up to a maximum of 8 days after the event. Standard deviations across events for each day after a rainfall day are shown as error bars (± 1 std). Linear regressions (*y* = *mx* + *b*) of the averaged *FC* with days after a rainfall event are conducted to test the sensitivity of CO_2_ fluxes to the storm event. We tested whether the slope of the linear regression (*m*) was significantly different from zero at *p* ≤ 0.05. Daily *FC* variations are sensitive to storm events only at the XL site (*m* = -1.59, Fig 10A), whereas the PL, ML, and REF sites have daily *FC* that is insensitive to precipitation (*m* of -0.06, 0.17, and -0.4 that are not significantly different from zero). This is consistent with the average *FC* differences between wet and dry days (Fig 9), but yields additional information on the rate of *FC* changes with time after a rainfall event, including insight on the transition from wet to dry days. Notably, the xeric landscaping with irrigated trees at XL had progressively more CO_2_ uptake (lower *FC*) as time progressed after rainfall events during the winter-spring. A similar behavior occurs at the REF site during the same period as XL (Fig 10B, *m* = -1.43, significantly different from zero), indicating that the CO_2_ uptake occurred across different urban landscapes and was likely tied to seasonal (winter-spring) conditions promoting a photosynthetic response of xeric trees. For instance, we visually noted that palo verde flowered after these winter-spring rainfall events. In contrast, the parking lot at PL exhibited a very small increase in CO_2_ emissions (higher *FC*) after rainfall events during the early summer that was also noted at REF (Fig 10B), but not at a significant level (mm byt *m* = 0.33). This suggests that additional water from precipitation during a period of high temperatures in May and June promotes CO_2_ efflux, mainly by increasing soil respiration in bare soil areas, in a similar fashion as noted in natural ecosystems of the region [90, 91, 99, 100]. During other times of the year, the site with ample outdoor water use (ML) does not respond to precipitation (*FC* remains the same), suggesting that a decoupling occurs between CO_2_ fluxes and storm inputs as commonly found in mesic regions.

## Conclusions

While bottom-up approaches have been used to estimate CO_2_ exchanges in arid and semiarid cities, few studies have carried out direct observations in different urban patch types. Indeed, a comparison of these approaches is warranted as the number of direct observations grows. At present, there are only a small number of studies discussing the controlling factors on *FC* and [CO_2_], such as vehicular emissions and plant photosynthetic activity, and their link to the proportion of these urban land covers within a site [for instance, 13, 15, 31, 35, 92, 94], though the temporal variation in vegetation and anthropogenic activity has typically not been taken into account to date. In this study, we conducted turbulent flux measurements using the EC technique to obtain a detailed view of CO_2_ fluxes and relate these to local meteorological conditions and urban characteristics for three short-term deployments and a stationary reference site in Phoenix, Arizona, USA. Comparisons to the suburban reference site were conducted during simultaneous periods for different seasons such that measured differences could be attributed to local variations in urban conditions. Results from the comparisons across the sites, seasons, and urban land cover types indicated the following:

1. Despite the small differences noted in meteorological conditions, the magnitude and behavior of *FC* and [CO_2_] varied considerably among the sites, in manners consistent with the urban land cover type. XL, PL, and REF acted as net sources of carbon dioxide, though plant activity was able to counteract anthropogenic emissions during mid-day periods. At ML, the well-watered turf grass was a net sink of CO_2_ during the summer season.
2. Diurnal variations in *FC* and [CO_2_] exhibited a strong correspondence to rush hour timing and vehicular counts for sites with large fractions of transportation surfaces, depending on local traffic behavior. Statistically significant differences were noted in *FC* between weekday and weekend days for all sites, except where vegetation activity served as a carbon dioxide sink. Vehicular emissions led to a temporal decoupling of CO_2_ and water vapor fluxes during the day.
3. Where urban irrigation supports a plant community, mid-day values in *FC* and [CO_2_] showed decreases consistent with the increase in measured latent heat flux. Statistically significant differences were noted in *FC* and [CO_2_] between sunny and cloudy days for most sites, except where the vegetation cover was low. A close correspondence was noted in the daily peak timing of CO_2_ and *ET* fluxes where outdoor water use supports plant photosynthesis.
4. The sensitivity of *FC* and [CO_2_] to precipitation events varied considerably among the sites in accordance with the proportion of irrigated vegetation. Where outdoor water use is abundant and frequent, CO_2_ conditions are insensitive to the occurrence of precipitation (wet versus dry days) or the time since the last rainfall event. This decoupling between CO_2_ fluxes and storm inputs suggests that irrigated landscapes in arid urban areas behave as mesic systems.

Based on these comparisons, key differences in the CO_2_ conditions can be attributed to the vegetation fraction and built surfaces in urban patches. Two of the sampled sites can be considered as end members that are dominated either by the effects of traffic and other anthropogenic emissions (PL) or by the carbon dioxide uptake from photosynthetic activities of turf grass and trees (ML). The other two sites (XL and REF) are characterized by combinations of these land cover types and thus exhibit intermediate or mixed behavior with respect to CO_2_ conditions. As noted by *Templeton et al*. [56], it would be desirable to conduct cross-site comparisons of this type over at a full year or longer to assess net effects of vehicular traffic and vegetation activity on CO_2_ fluxes. Such a study could also quantify the seasonal variations in these factors responding to plant phenology and temporal changes in anthropogenic activities. For instance, the role played by seasonality and its interaction with irrigation is considered important in determining if plant activity can fully counteract anthropogenic CO_2_ emissions during an annual period. For the periods studied here, vegetation could not counteract CO_2_ emissions, leading to a net carbon dioxide source at all sites, including the mesic landscaping. Nevertheless, this cross-site comparison suggests a fruitful avenue for scaling up CO_2_ conditions to larger areas by using the fraction occupied by urban vegetation and built surfaces. Following this strategy could lead to considerable improvements in bottom-up estimates of CO_2_ fluxes and concentrations to better capture the anticipated spatiotemporal variability in desert cities.
